# Challenges in Diagnosis and Management of Takayasu Arteritis: A Case Report Highlighting Vascular Complications and Delayed Recognition

**DOI:** 10.1002/ccr3.70174

**Published:** 2025-02-05

**Authors:** Bindira Adhikari, Biraj Niraula, Polina Dahal, Anil Suvedi, Gaurav Subedi

**Affiliations:** ^1^ Chitwan Medical College Bharatpur Nepal

**Keywords:** cardiovascular disorder, chronic disease, ophthalmology, vascular surgery

## Abstract

Takayasu arteritis is a rare systemic inflammatory condition that primarily affects medium and large arteries, mainly in young Asian women with an estimated incidence of 1–2 per million. Diagnosis is challenging due to nonspecific early symptoms, resulting in an insidious clinical course until vascular complications arise. A 30‐year female presented with progressive easy fatigue over 4 years. She developed recent visual disturbances, headaches, and dizziness. Examination revealed unrecordable blood pressures and vascular bruits. Imaging revealed vascular involvement including stenosis of arch of aorta and its all branches. The available treatments can ease symptoms and slow progression, but the late diagnosis caused lifelong morbidity, with no procedure to resolve persistent fatigue. TA poses diagnostic challenges due to its nonspecific early symptoms and risk of severe vascular complications. This case highlights the importance of considering TA in young women with vague systemic inflammatory symptoms.

## Graphical abstract

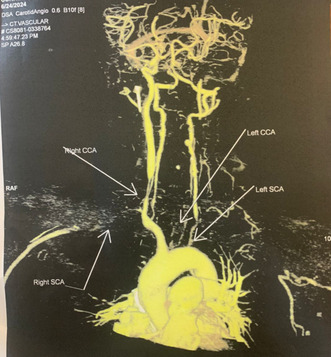

**Summary**

- Takayasu arteritis can start with vague symptoms like upper limb fatigue, complicating early diagnosis.
- This case highlights the critical need for suspicion in young women with such symptoms to prevent severe progression and significant impacts on quality of life.

## 1 Introduction

Takayasu arteritis (TA), also known as pulseless disease, is a rare systemic inflammatory condition that primarily affects the medium and large arteries, including their branches. It predominantly occurs in young Asian women, with a reported worldwide incidence of only 1 to 2 per million people [1].

Women are more frequently affected than men [2], with varying incidence rates in different parts of the globe. Male to female ratio is 1:8 in Western countries and 1:3 in Japan [3].

It is a chronic disease primarily impacting the aorta and its large branches. Early diagnosis is crucial to preventing severe end organ damage, including stroke and ischemic heart disease [4].

However, diagnosis is often challenging due to the non‐specific systemic inflammatory symptoms present in the early phase, which can lead to an insidious clinical course until vascular ischemic complications emerge [5].

The disease typically progresses through two phases: an initial pre‐occlusive inflammatory phase that may go unnoticed, followed by an occlusive phase characterized by ischemic vascular symptoms resulting from arterial lesions such as stenosis, occlusion, or aneurysm [6].

Extremity pain, claudication, bruits, pulselessness and unrecordable blood pressure are the common features of patient visiting health care facility. However, presentation with acute visual loss or stroke may be particularly rare [7].

18% of patients with large vessel vasculitis presents with unilateral visual loss at diagnosis, often resulting in irreversible damage. Early administration of pulsed intravenous methylprednisolone may provide some benefit to patients experiencing early onset of visual symptoms [8].

While numerous systematic reviews have explored ocular manifestations in various systemic diseases, few have focused on the eye involvement in Takayasu arteritis. This report emphasizes the importance of early diagnosis, the varied and subtle presentation of the disease, and the long‐term challenges faced by those with TA.

## 2 Case History/ Examination

A 30‐year female weighing 59 kg presented 3 months back with a 4‐year history of easy fatigue in the bilateral upper limbs, initially triggered by strenuous activity. She was diagnosed with anemia and multivitamin deficiency and managed accordingly. However, fatigue progressively worsened over the last 7 days and is now present even on general activities. Recently, she also experienced limb claudication, jaw claudication while eating, bilateral temporal headaches with orbital pain, and painful, intermittent blurred vision, which worsened over the last 3 days. Additionally, she reported dizziness and lightheadedness for the past 3 days.

On examination, her systolic blood pressure was 60 mmHg with an unrecordable diastolic pressure; her respiratory rate was 24 breaths per minute, her pulse was 97 beats per minute (bpm), her temperature was 97°F, and her oxygen saturation was 98% on room air. She had a Glasgow Coma Scale (GCS) score of 15/15, and her random blood sugar was 150 mg/dL. Physical examination revealed pallor in the bilateral palpebral conjunctiva, a feeble pulse in the right radial artery with no pulse in the left radial artery, palpable bilateral dorsalis pedis, a bruit over the right carotid artery, and absent bruit over the left. Systemic examinations were otherwise normal. There was no similar history in the family.

## 3 Methods (Investigations and Treatment)

Blood investigations showed a leukocyte count of 8320/mm^3^ (normal range: 4000‐11,000/mm^3^), platelets at 288,000/mm^3^ (150,000‐400,000), RBC at 4.25 million/mm^3^ (4–5 million/mm^3^), hemoglobin at 10 mg/dL (12–16 mg/dL for women), mean cell volume at 76.2 fl (80–99 fl), mean cell hemoglobin concentration at 30.9 g/dL (32–36 g/dL), mean cell hemoglobin at 23.6 pg. (26–32 pg), packed cell volume at 32.4% (36%–54%), erythrocyte sedimentation rate at 66 mm/h (0–20 mm/h for < 50 years female), and C‐reactive protein at 22.9 mg/L (0–5 mg/L). Serum creatinine was 0.66 mg/dL (0.4–1.4 mg/dL), sodium was 140 mmol/L (135–150 mmol/L), and potassium was 4.11 mmol/L (3.5–5.5 mmol/L). Thyroid and liver function tests were normal. Urine examination revealed plenty of pus cells with positive (++) leukocyte esterase, but no growth in urine culture. Abdominal and pelvic ultrasound scans were normal and advised follow up.

Cardiothoracic consultation was done suspecting cardiovascular disorder which advised carotid CT angiogram. The patient was given two rapid boluses of 500 mL Ringer's Lactate (RL) over an hour. Still, the blood pressure could not be recorded in the upper limb; however it was 130/80 mm of Hg in the bilateral lower limbs. The blood pressure fluctuation is shown below in Table 1. A pint of RL was then infused at 30 mL/h, maintaining stable vitals.

**TABLE 1:** BP fluctuations of the patient.

Day 0	At 11: 40 AM	60/−	Upper limb blood pressure
	At 12:15 PM	130/80	Lower limb blood pressure
	At 1 PM	170/100	
	At 2 PM	150/90	
	At 3 PM	160/90	
	At 4 PM	170/90	
	At 5 PM	120/80	
	At 6 PM	130/70	
	At 7 PM	170/70	
	At 8 PM	180/100	
Day 1	At 2 PM	110/80	
Day 2	At 1 PM	128/80	
Day 3	At 12 PM	130–120/80–90	

She was kept on tablet Aspirin 75 mg per oral once a day (OD), injection methylprednisolone, nitrofurantoin 100 mg OD, pantoprazole 40 mg per oral OD and other supportive measures.

On her first day of admission (DOA), ophthalmology consultation was done. It showed the following visual acuity (Table 2) results:

**TABLE 2:** Visual acuity.

Visual acuity	Right eye	Left eye
Unaided	6/9 partial	6/18 partial
Pin hole	6/6 partial	6/9

**FIGURE 1:**
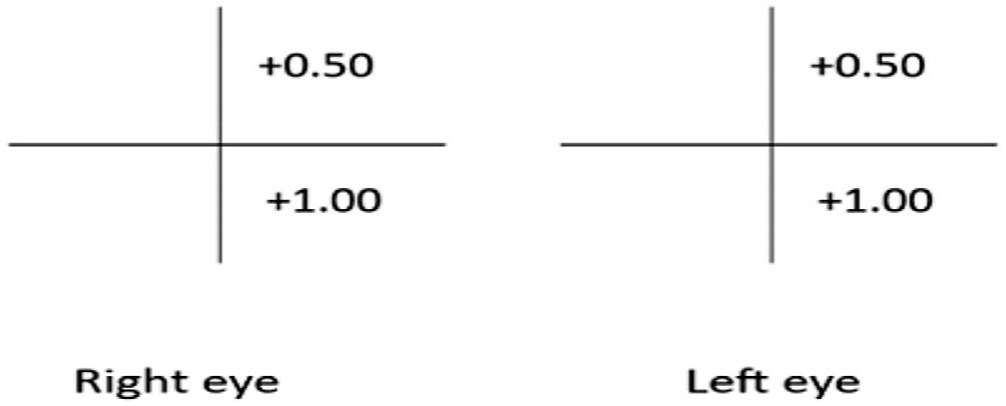
Retinoscopy findings of both eyes.

Eyeball examination was normal, and all duction and version movements were within normal range. Posterior segment evaluation showed a cup‐disc ratio of 0.3:1, arteriovenous ratio of 2:4, positive foveal reflex, bilateral microaneurysms, and flame‐shaped hemorrhages more in the peripheral retina. Retinoscopy at 66 cm (Figure 1) indicated a correction of −0.50D cylindrical lens at 110° for the right eye (6/6 partial) and − 0.50D spherical lens with −0.50D cylindrical lens at 90° for the left eye (6/9). The patient was kept under observation for blurred vision.

**FIGURE 2:**
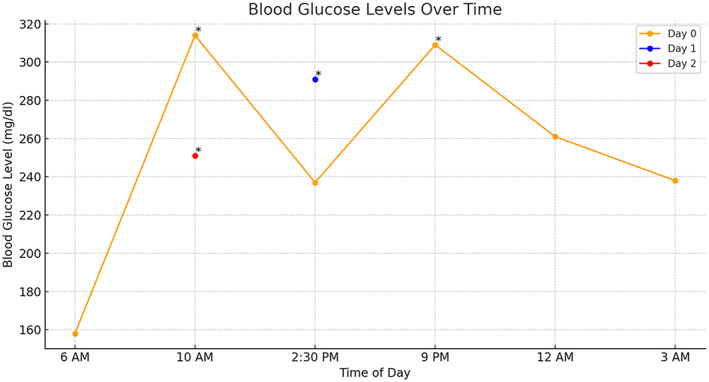
Line chart showing blood glucose fluctuations and regular insulin administration.

The patient was well and alert with no new issues. However, she was measured hyperglycemic throughout the day as shown below in line graph (Figure 2) which prompted to manage with injection regular insulin 6 unit (indicated as *). The hyperglycemic episodes were attributed to extensive corticosteroid administration.

**FIGURE 3:**
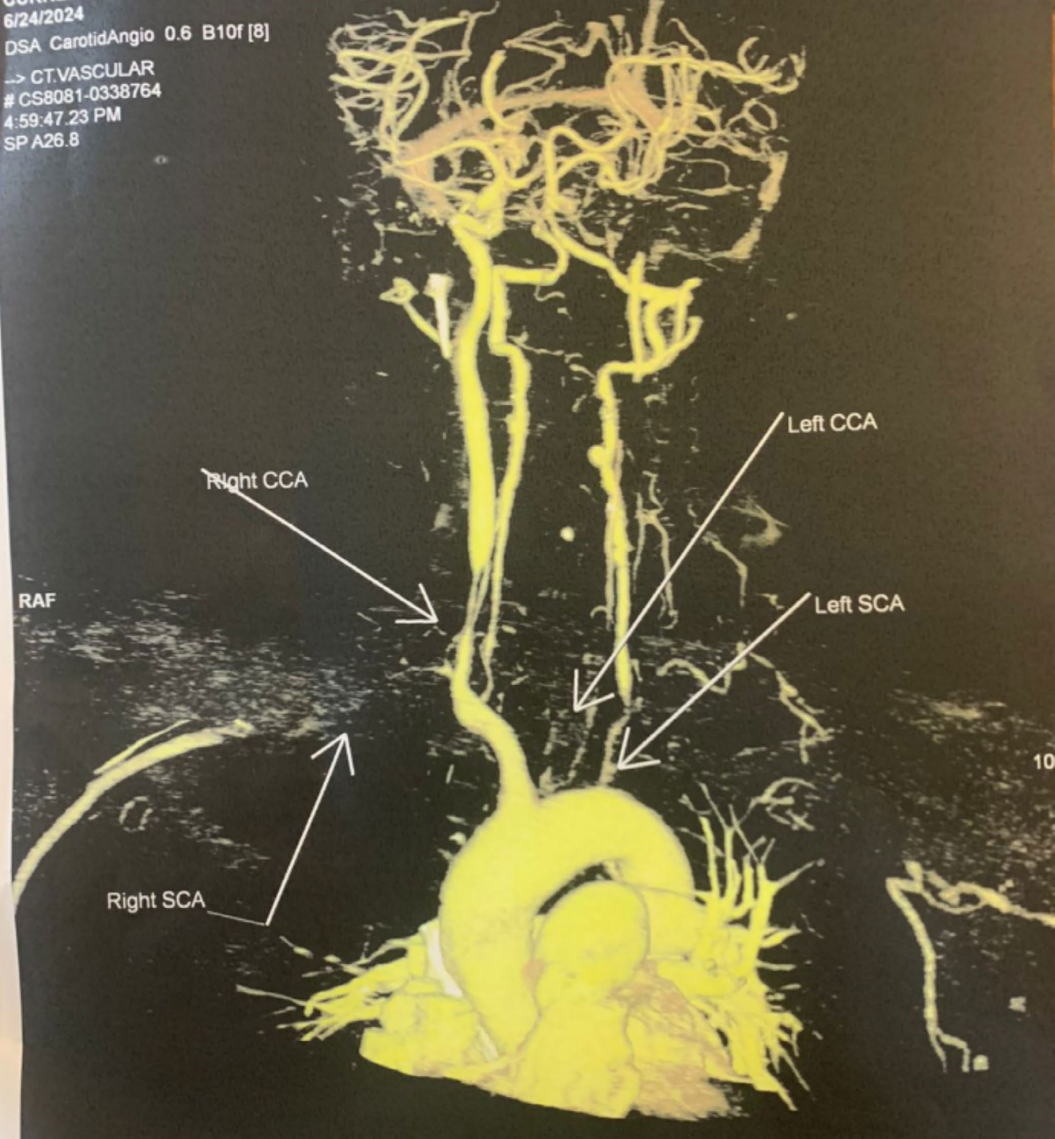
Carotid CT angiogram demonstrating circumferential thickening of all branches of arch of aorta.

**FIGURE 4:**
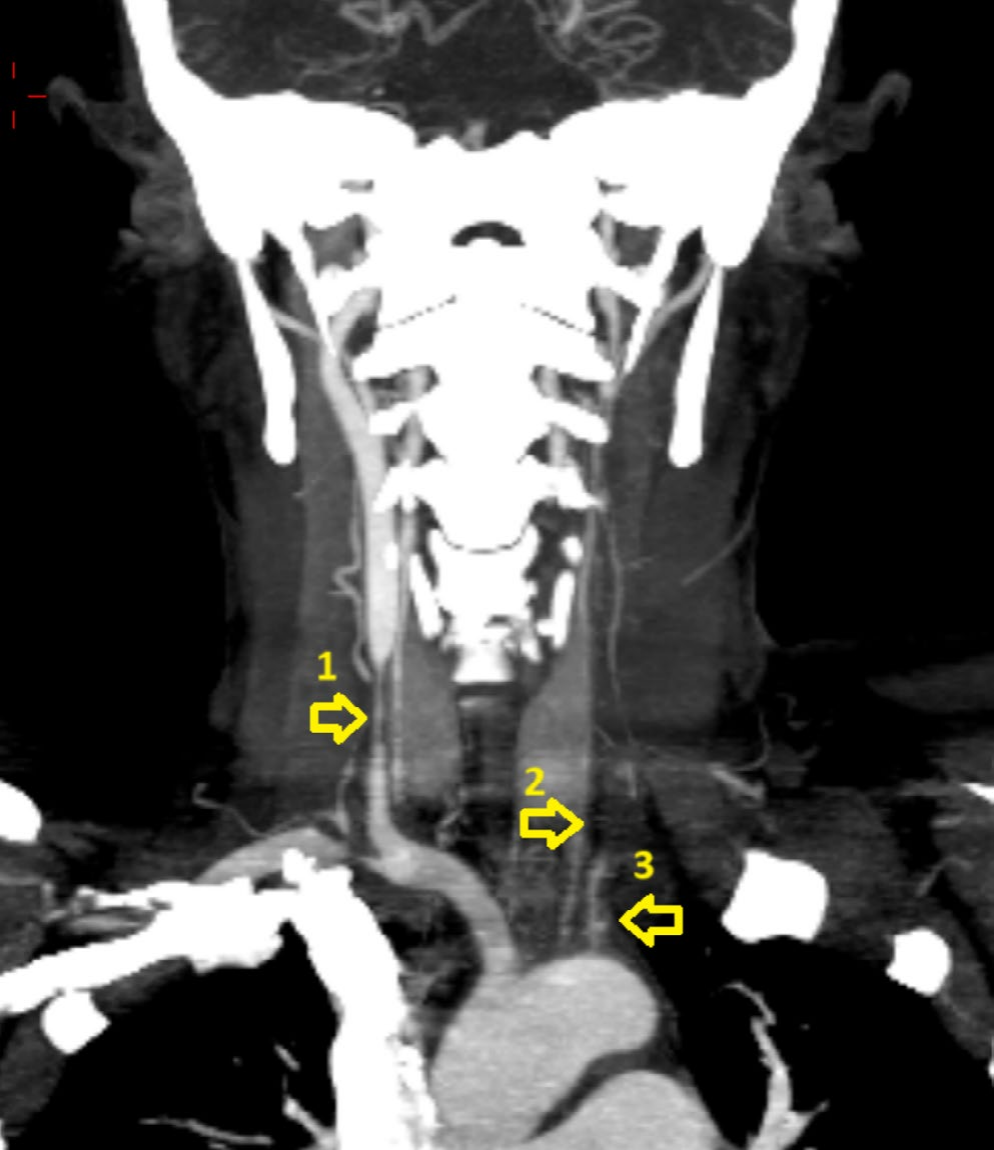
CT‐ Coronal view demonstrating stenosis of all arteries arising from arch of aorta as indicated by arrows. 1: Stenosis of right common carotid artery with post‐stenotic dilation, 2: Left common carotid artery stenosis along its entire course, 3: Left sub‐clavian artery stenosis.

**FIGURE 5:**
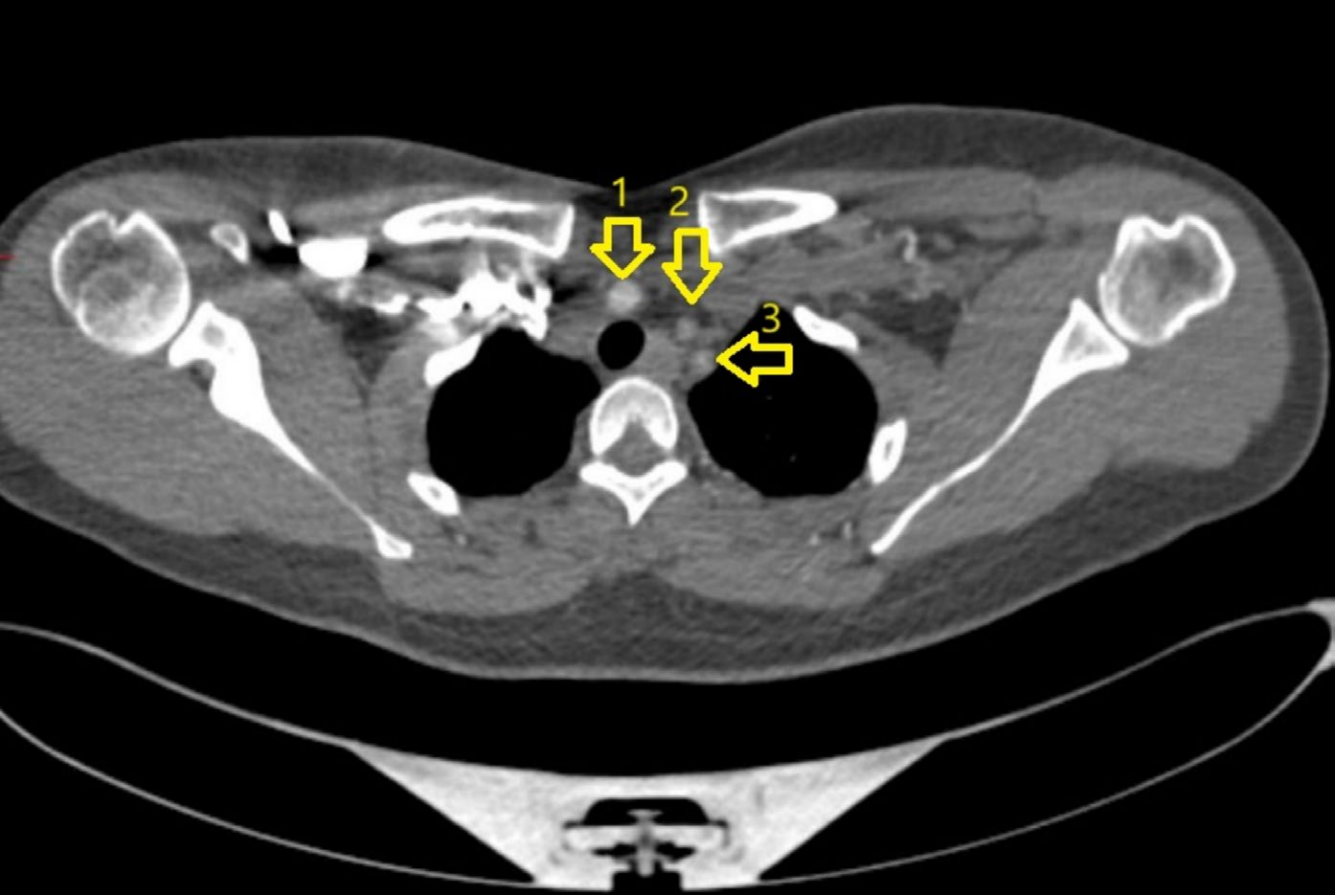
CT‐ axial view demonstrating stenosis of brachiocephalic trunk [1], left common carotid [2] and left sub‐clavian artery [3].

On her second DOA, Carotid CT angiogram revealed features likely of type 1 Takayasu arteritis with following findings (Figure 3). There was circumferential symmetric thickening of arch of aorta and it is all branches with left common carotid artery stenosis by 90% in its entire course. The above CT findings of the patient is demonstrated in Figures 4 and 5 respectively. Patient BP was monitored and planned to add amlodipine 2.5 mg per oral OD diagnosing reflex hypertension. CTVS recommended steroid treatment for the initial 2 weeks followed by bypass channel formation between right common carotid artery and right brachiocephalic trunk or arch of aorta.

She was planned to give induction with high‐dose methylprednisolone (1 g) for three doses in 3 days followed by tablet prednisolone (60 mg per oral OD) and Azathioprine (50 mg per oral OD) with a plan to increase azathioprine to 50 mg twice a day (BD) and taper dose of prednisolone in next 2 weeks after baseline complete blood counts monitor. She was also prescribed tablet folic acid (5 mg, five times a week), tablet aspirin (75 mg), tablet rosuvastatin and tablet cefuroxime and clavulanic acid (500 mg). Regular insulin was administered as per requirement dictated by blood glucose level. The use of antibiotics was justified for focus of infection seen in routine urine examination.

On the fourth DOA, the patient was discharged with hemodynamically stable vitals: pulse 78 bpm, BP 120/80 mmHg at bilateral lower limb, respiratory rate 20 breaths per minute, temperature 97.3°F, and oxygen saturation 98% on room air.

## 4 Conclusion and Results (Outcome)

The delayed diagnosis resulted in lifelong morbidity for the patient, with no procedure able to resolve the persistent fatigue experienced. However, she was counseled to undergo arterial bypass which would relief dizziness temporarily.

## 5 Discussion

Large vessel vasculitis, particularly the aorta and its branches, is the most prevalent form of primary vasculitis in adults. This includes giant cell arteritis (GCA) and Takayasu arteritis, both types of granulomatous arteritis are distinguished by their patterns of vessel involvement. GCA is the most common primary vasculitis globally, predominantly affecting individuals over 50, with incidence rates peaking in the eighth decade of life [9].

In contrast, Takayasu arteritis usually manifests in the second or third decade of life [7].

Global incidence rates of GCA show considerable variation, with Northern Europe reporting as high as 44 cases per 100,000 people over 50, while Southern Asia reports about 0.3 cases per 100,000 [9].

Similarly, Takayasu arteritis has a higher incidence in regions like Asia, Africa, and Latin America, with Asia showing an incidence rate of 2.69 cases per million per year—approximately 100 times higher than Europe and North America [7]. It primarily affects young females in their 20 and 30, though cases have been documented in children as young as 24 months [10].

The aorta and large conducting arteries are considered immune‐protected, generally safeguarded from inflammatory attacks. However, a breach in this immune‐protection can lead to autoimmune vasculitis, as seen in GCA, where CD8+ T regulatory cells fail to control CD4+ T cells and macrophages, resulting in destructive granulomatous lesions [11].

This loss of immune tolerance is also significant in Takayasu arteritis [9], which progresses with fibrotic narrowing of the aorta and its major branches, potentially leading to complications such as vessel narrowing, clot formation, dilation, or aneurysm formation [7].

Its progression involves the gradual development of fibrotic narrowing in the aorta and its major branches. This can lead to various complications such as narrowing, clot formation, dilation, or even the formation of aneurysms [7].

According to Sharma et al.'s study, Indian and South Asian patients more frequently exhibit Type II and III angiographic involvement compared to Type I, which contrasts with the predominant Type I involvement seen in Japan and Western countries. Japanese patients typically experience the disease starting in the ascending aorta and arch before progressing to the thoraco‐abdominal aorta. In contrast, Indian patients commonly initially present with involvement of the abdominal aorta, which then progresses upward to affect the thoracic aorta [3]. It often presents with varied clinical manifestations. In our patient, the presenting symptoms developed lately were painful blurring of vision and orbital pain with an ocular examination finding of 2:4 arteriovenous ratio indicative of venous engorgement which align closely with recognized ophthalmic manifestations of Takayasu arteritis (TAK) [12].

It has an insidious onset, which manifests as arteritis early in the course resulting in segmental stenosis, occlusion, dilatation and/or aneurysm of the vessel [7]. Vessel wall thickening, narrowing, and complete blockage can lead to reduced blood flow and tissue damage, posing significant risks to individuals with Takayasu's arteritis (TA). This condition can impair organ function due to ischemia, potentially threatening the lives of affected patients. In addition, involvement of the coronary arteries in TA is particularly concerning, as it is associated with a poor prognosis and higher mortality rates. Furthermore, performing coronary artery revascularization in patients with active TA is challenging and carries an elevated risk of major adverse cardiac events (MACE) [13].

A case of type III TA reported by Del et al. in a 25 year female with 7 years of prednisolone administration resulted in disease remission, control and also improves the diameter of abdominal aorta [14].

EULAR guidelines suggested Prednisolone as the primary treatment choice with an initial dosage of 1 mg/kg/day (up to a maximum of 60 mg/day) maintaining for a month, followed by a gradual reduction in dose. In most cases, additional immunosuppressive therapy is necessary to reduce the risk of steroid‐related side effects and manage disease progression. It's crucial to note that discontinuing steroid treatment can often lead to relapses. Up to 70% of Takayasu arteritis patients may require vascular surgery or bypass grafting to address aspects like renovascular hypertension. Although it has a favorable outcome, subsequent revision surgery are often necessary. Angioplasty and stent placement are associated with higher restenosis rates. It is advisable to schedule elective procedures during periods of disease remission requiring Long‐term follow‐up [8].

Similarly, tocilizumab (TCZ) stood better than traditional DMARDs in patient involving coronary arteries with TA in terms of reducing disease activity as a whole, improving lumen stenosis and reduction of glucocorticoid dose post‐ TCZ treatment for 6 months [13].

Kwon et al. revealed that the administration of statins on TAK patients with active disease substantially reduces the relapse rate following remission attainment in this population [15].

In our case, 4 years after the onset of the disease, imaging studies revealed extensive vascular changes, including 90% stenosis of the left common carotid artery and complete occlusion of the left subclavian artery with distal reformation. The delayed diagnosis resulted in lifelong morbidity for the patient, with no procedure being able to resolve the persistent fatigue she experienced. However, she was counseled to undergo arterial bypass which would relief dizziness temporarily. As the disease progresses beyond the bypass's effectiveness, the symptoms would reappear. Despite treatment initiation with high‐dose steroids and immunosuppressants, the disease had progressed significantly, limiting treatment options to symptomatic relief and delay of further progression which profoundly impacted on her quality‐adjusted life year. Study has shown that, it significantly impacts patients' quality of life, with both physical and mental health scores lower than those of many other chronic diseases involving peripheral vascular disease [16]. This case shows the typical natural history of type 1 Takayasu arteritis and the dreadful morbidity the patient had to suffer.

## Author Contributions

**Bindira Adhikari:** conceptualization, writing – original draft, writing – review and editing. **Biraj Niraula:** validation, writing – original draft, writing – review and editing. **Polina Dahal:** conceptualization, writing – review and editing. **Anil Suvedi:** writing – review and editing. **Gaurav Subedi:** writing – review and editing.

## Consent

Written informed consent was obtained from the patient for the publication of this case report and accompanying images. A copy of the written consent is available for review upon request.

## Conflicts of Interest

The authors declare no conflicts of interest.

## Data Availability

Data available on request from the authors.

## References

[1] B. Trinidad , N. Surmachevska , and V. Lala , “Takayasu Arteritis,” in StatPearls [Internet] (StatPearls Publishing, 2023). 29083666

[2] F. Onen and N. Akkoc , “Epidemiology of Takayasu Arteritis,” Presse Médicale 46, no. 7–8 (2017): e197–e203. 28756072 10.1016/j.lpm.2017.05.034

[3] S. Sharma , K. Sangameswaran , and S. Kalra , “Clinical Profile of TAKAYASU'S Arteritis,” Medical Journal, Armed Forces India 54, no. 2 (1998): 140–142. 28775449 10.1016/S0377-1237(17)30505-1 PMC5531315

[4] A. Somashekar and Y. T. Leung , “Updates in the Diagnosis and Management of Takayasu's Arteritis,” Postgraduate Medicine 135, no. sup1 (2023): 14–21. 36588528 10.1080/00325481.2022.2159723

[5] M. L. F. Zaldivar Villon , J. A. L. de la Rocha , and L. R. Espinoza , “Takayasu Arteritis: Recent Developments,” Current Rheumatology Reports 21, no. 9 (2019): 45. 31321560 10.1007/s11926-019-0848-3

[6] M. Maz , S. A. Chung , A. Abril , et al., “2021 American College of Rheumatology/Vasculitis Foundation Guideline for the Management of Giant Cell Arteritis and Takayasu Arteritis,” Arthritis and Rheumatology 73, no. 8 (2021): 1349–1365. 34235884 10.1002/art.41774 PMC12344528

[7] N. Setty , “Takayasu's Arteritis ‐ a Comprehensive Review,” Journal of Rare Diseases Research & Treatment 2, no. 2 (2017): 63–68.

[8] C. Mukhtyar , L. Guillevin , M. C. Cid , et al., “EULAR Recommendations for the Management of Large Vessel Vasculitis,” Annals of the Rheumatic Diseases 68, no. 3 (2009): 318–323. 18413441 10.1136/ard.2008.088351

[9] D. Pugh , M. Karabayas , N. Basu , et al., “Large‐vessel vasculitis,” Nature Reviews Disease Primers 7, no. 1 (2022): 93. 10.1038/s41572-021-00327-5 PMC9115766 34992251

[10] N. Cakar , F. Yalcinkaya , A. Duzova , et al., “Takayasu Arteritis in Children,” Journal of Rheumatology 35, no. 5 (2008): 913–919. 18381778

[11] K. Jin , Z. Wen , B. Wu , et al., “NOTCH‐Induced Rerouting of Endosomal Trafficking Disables Regulatory T Cells in Vasculitis,” Journal of Clinical Investigation 131, no. 1 (2021): e136042. 32960812 10.1172/JCI136042 PMC7773364

[12] S. Mahajan , U. C. Behera , S. L. Pravabati , M. Shah , S. K. Padhy , and A. Kelgaonkar , “Pulseless and Blindness–an ophthalmologist's Role in Diagnosing Takayasu Arteritis: Case Series and Brief Review of Literature,” European Journal of Ophthalmology 31, no. 6 (2021): 3525–3531. 33579172 10.1177/1120672121990577

[13] L. Pan , J. Du , J. Liu , et al., “Tocilizumab Treatment Effectively Improves Coronary Artery Involvement in Patients With Takayasu Arteritis,” Clinical Rheumatology 39, no. 8 (2020): 2369–2378. 32144625 10.1007/s10067-020-05005-7

[14] L. Del Corso , D. Moruzzo , M. Agelli , and F. Pentimone , “Takayasu's Arteritis on Steroid Therapy. Seven Years Follow‐Up,” Panminerva Medica 41, no. 4 (1999): 355–358. 10705719

[15] O. C. Kwon , J. S. Oh , M. C. Park , et al., “Statins Reduce Relapse Rate in Takayasu Arteritis,” International Journal of Cardiology 287 (2019): 111–115. 30824260 10.1016/j.ijcard.2019.02.046

[16] C. J. Abularrage , M. B. Slidell , A. N. Sidawy , P. Kreishman , R. L. Amdur , and S. Arora , “Quality of Life of Patients With Takayasu's Arteritis,” Journal of Vascular Surgery 47, no. 1 (2008): 131–137. 18178464 10.1016/j.jvs.2007.09.044

